# Maturation Mechanism of Nitrile Hydratase From *Streptomyces canus* CGMCC 13662 and Its Structural Character

**DOI:** 10.3389/fmicb.2020.01419

**Published:** 2020-06-25

**Authors:** Ling Guo, Xi Cheng, Huo-Yong Jiang, Yi-Jun Dai

**Affiliations:** Jiangsu Key Laboratory for Microbes and Functional Genomics, Jiangsu Engineering and Technology Research Center for Industrialization of Microbial Resources, College of Life Science, Nanjing Normal University, Nanjing, China

**Keywords:** nitrile hydratase, maturation mechanism, cobalt insertion, self-subunit swapping, substrate specificity, protein–ligand interaction

## Abstract

Nitrile hydratases have received significant interest both in the large-scale industrial production of acrylamide and nicotinamide, and the remediation of environmental contamination with nitrile-containing pollutants. Almost all known nitrile hydratases include an α-subunit (AnhA) and β-subunit (AnhB), and a specific activator protein is crucial for their maturation and catalytic activity. Many studies exist on nitrile hydratase characteristics and applications, but few have reported their metal insertion and post-translational maturation mechanism. In this study, we investigated the cobalt insertion and maturation mechanism of nitrile hydratase from *Streptomyces canus* CGMCC 13662 (*Sc*NHase) bearing three subunits (AnhD, AnhE, and AnhA). *Sc*NHase subunits were purified, and the cobalt content and nitrile hydratase activity of the *Sc*NHase subunits were detected. We discovered that cobalt could insert into the cobalt-free AnhA of *Sc*NHase in the absence of activator protein under reduction agent DL-dithiothreitol (DTT) environment. AnhD not only performed the function of AnhB of NHase, but also acted as a metal ion chaperone and self-subunit swapping chaperone, while AnhE did not act as similar performance. A cobalt direct-insertion under reduction condition coordinated self-subunit swapping mechanism is responsible for *Sc*NHase post-translational maturation. Molecular docking of *Sc*NHase and substrates suggested that the substrate specificity of *Sc*NHase was correlated with its structure. *Sc*NHase had a weak hydrophobic interaction with IAN through protein–ligand interaction analysis and, therefore, had no affinity with indole-3-acetonitrile (IAN). The post-translational maturation mechanism and structure characteristics of *Sc*NHase could help guide research on the environmental remediation of nitrile-containing waste contamination and three-subunit nitrile hydratase.

## Introduction

**FIGURE 1:**
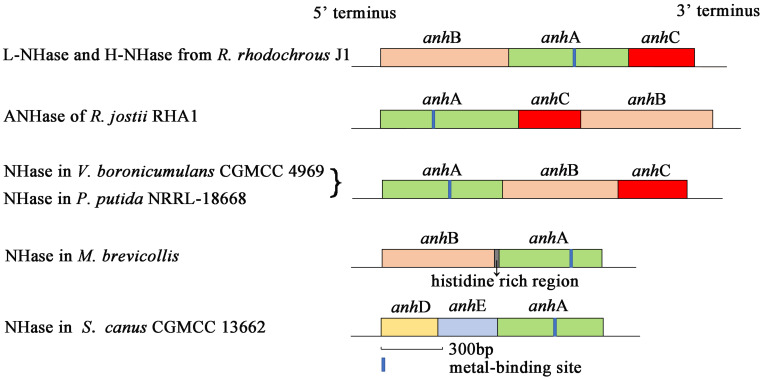
Schematic representation of five NHases with different gene organizations.

Nitrile hydratase (NHase; EC 4.2.1.84) catalyzes the hydration of nitriles to give the corresponding amides (R–CN + H_2_O → R–CONH_2_) (Kobayashi et al., 1992; Yamada and Kobayashi, 1996; Kobayashi and Shimizu, 1998). NHase is a metalloenzyme composed of an α-subunit (AnhA) and β-subunit (AnhB). The subunit gene organization of some previously reported NHases are shown in Figure 1. An activator protein gene (*anh*C) is necessary for NHase from *Variovorax boronicumulans* CGMCC4969 (*Vb*NHase), NHase from *Pseudomonas putida* NRRL-18668 (*Pp*NHase), L-NHase and H-NHase from *Rhodococcus rhodochrous* J1, and ANHase from *Rhodococcus jostii* RHA1 structural genes to achieve functional expression (Liu et al., 2012, 2013; Odaka and Kobayashi, 2013; Sun et al., 2017). A conserved metal-binding motif (Cys-X(Thr/Ser)-Leu-Cys-Ser-Cys) is found in all known Co-type and Fe-type NHase AnhA subunits. Most evidence has emphasized that the post-translational modification of cysteine into cysteine-sulfinic acid (αCys-SO_2_H) and cysteine-sulfenic acid (αCys-SOH) is essential for catalytic efficiency (Nagashima et al., 1998; Murakami et al., 2000; Miyanaga et al., 2001; Hourai et al., 2003; Martinez et al., 2014). This metal-binding motif is known as the metal center of the metalloenzyme. Some investigators, particularly biomimetic inorganic chemists, have focused on the mechanisms of biological metallocenter assemblies in nature to obtain useful synthetic insight. Metallocenter biosynthesis mechanisms can be summarized as follows (Kuchar and Hausinger, 2004): (i) Reversible metal-ion binding; (ii) synergistic binding of metal with another component; (iii) metallochaperone delivery of metal ion or cofactor; (iv) post-translational modification to create a metal-binding site; (v) synthesis of metal-containing cofactors; (vi) requirement of an apoprotein-specific molecular chaperone; and (vii) metal incorporation coupled with electron transfer.

Although NHases have been characterized by researchers for decades, only a few reports have focused on the NHase metallocenter assembly or maturation mechanism. The activator-dependent maturation mechanism of Co-type NHase has been discovered and a self-subunit swapping model was proposed (Zhou et al., 2008, 2009, 2010; Liu et al., 2012). The post-translational maturation of L-NHase and H-NHase from *R. rhodochrous* J1, NHase from *P. putida* NRRL-18668, and ANHase from *R. jostii* RHA1 relies on self-subunit swapping (Zhou et al., 2009, 2010; Okamoto et al., 2010; Liu et al., 2012). Self-subunit swapping is a type of post-translational modification maturation in which metal ion insertion occurs at the target subunit of a multisubunit metalloprotein. This process requires a metallochaperone (M), generally known as an activator protein, which is essential for cobalt incorporation into the active center and self-subunit swapping. Zhou et al. (2009) found that cobalt insertion into apoprotein (Apo)-L-NHase was dependent on NhlE *in vitro*. NhhG participated as a metal chaperone in cobalt incorporation in H-NHase (Zhou et al., 2010). P14K, as a metallochaperone, was shown to be involved in cobalt incorporation in NHase from *P. Putida* NRRL-18668 (Liu et al., 2012). The features of self-subunit swapping can be summarized as follows: (i) The metallochaperone does not deliver cobalt to the Apo-AnhA, but enables AnhA to incorporate cobalt; (ii) cobalt first inserts into the AnhAM_2_ complex; (iii) the Co-bounded AnhAM_2_ complex named as holoprotein (Holo)-AnhAM_2_ possess cysteine-oxidized AnhA; (iv) subunit swapping occurs between an unmodified AnhA and modified AnhA; and (v) self-subunit swapping is a subunit-specific reaction. Importantly, in contrast to all previously reported NHases, structural genes of *Sc*NHase have the order <*anh*D><*anh*E><*anh*A> (Figure 1), with *anh*D and *anh*E identified as β-subunit partial peptides (Guo et al., 2019). No *anh*C was recognized in the *Sc*NHase genome, and *Sc*NHase is similar to eukaryotic NHase (Guo et al., 2019). Therefore, investigating the post-transitional maturation of *Sc*NHase represents a meaningful and interesting study.

Researchers have applied NHase from *R. rhodochrous* J1, *R. jostii* RHA1, *P. putida* NRRL-18668, and *M*. *brevicolli*s to the transformation of nitriles, such as acrylonitrile, 3-cyanopyridine, acetonitrile, and benzonitrile (Okamoto and Eltis, 2007; Zhou et al., 2009; Liu et al., 2012; Martinez et al., 2017). However, *Vb*NHase and *Sc*NHase in our laboratory have been applied to remediate environments contaminated by toxic nitrile-containing waste, particularly neonicotinoid pesticides used extensively worldwide, such as acetamiprid (ACE) and thiacloprid (THI) (Zhou et al., 2014; Sun et al., 2016; Guo et al., 2019). Meanwhile, *Vb*NHase from plant-growth-promoting rhizobacteria *V. boronicumulans* CGMCC 4969 can produce indole-3-acetamide (IAM), the precursor of phytohormone indole-3-acetic acid (IAA), using indole-3-acetonitrile (IAN), which engages in interactions between microbes and plants in a mutually beneficial manner (Sun et al., 2018). In contrast, *Sc*NHase has shown almost no activity toward IAN (Guo et al., 2019). Therefore, we investigated the *Sc*NHase structural character to explore substrate specificity. In this study, we investigated the post-translational maturation mechanism of *Sc*NHase and proposed a possible maturation mechanism model for three-subunit NHase for the first time. Interestingly, we found that cobalt ion independent on activator protein could insert into AnhA of apoenzyme *Sc*NHase in the presence of DTT, and Holo-AnhDA could activate apoenzyme *Sc*NHase to yield holoenzyme. The substrate specificity of *Sc*NHase was correlated with its structural character. These results aid the biodegradation of toxic nitrile-containing contaminates and provide a theoretical basis for the maturation mechanism of three-subunit NHase and eukaryotic NHase.

## Materials and Methods

### Chemicals and Media

ACE (>98% purity) was provided by Dr. Haijun Ma of the Jiangsu Pesticide Research Institute, Nanjing, China. A standard cobalt solution (100 μg/mL in 1% HNO_3_) was purchased from Aladdin Biochemical Technology Co., Ltd. (Shanghai, China). Other chemicals were purchased from Sangon Biotech (Shanghai, China).

Lysogeny broth medium (LB; pH 7.2), used to incubate bacteria, was prepared by dissolving yeast extract (5.0 g), peptone (10.0 g), and NaCl (10.0 g) in deionized water (1.0 L).

### Strains and Plasmid Construction

Strains *E. coli*-Q0, Q1, Q2, Q3, and Q4 were constructed as described in our previous study. Plasmids Q5 and Q6 were constructed in vector pET-21a(+). A SanPrep Column Plasmid Mini-Preps Kit was used to extract plasmids. TaKaRa MiniBEST Bacteria Genomic DNA Extraction Kit Ver. 3.0 was used to extract the bacteria genome. TaKaRa MiniBEST Agarose Gel DNA Extraction Kit Ver. 4.0 was used to purify the target genes after amplification. To investigate the function of AnhD and AnhE, co-expression strain *E. coli*-Q5 was constructed by introducing plasmids Q4 and Q5 together into *E. coli* Rosetta competent cells, while strain *E. coli*-Q6 was constructed by introducing plasmids Q4 and Q6 together into *E. coli* Rosetta competent cells. The primers used to construct plasmids in this study are shown in Table 1. A ClonExpress II one-step cloning kit (Vazyme Biotech, Nanjing, China) was used to construct recombinant plasmids. The recombinant strains were then verified by DNA sequencing (Sipkin Biotech, Nanjing, China).

**TABLE 1:** Primers used to construct plasmids^*a*^.

**Target gene**	**Primers**	**Sequence (5′-3′)**
*anh*D	F1	ACAGCAAATGGGTCGCGGATCC**GAATTC**AT GGCCAGGATCAACGACGTCGG
	R1	ATCTCAGTGGTGGTGGTGGTGGTG**CTCGAG** TCAGTCATCCAGCTCACCCGGTTCG
*anh*E	F2	ACAGCAAATGGGTCGCGGATCC**GAATTC**AT GACTGACCGTTTCCCGCC
	R2	ATCTCAGTGGTGGTGGTGGTGGTG**CTCGAG** CCGCTCATCTTCACGGTC

*^*a*^Bases in bold (GAATTC and CTCGAG) are restriction enzyme sites of *EcoR*I and *XhoI*, respectively.*

### Protein Expression and Purification

Recombinant plasmids were delivered into *E. coli* Rosetta competent cells by calcium chloride transformation, with the resulting transformants, including target plasmids, verified by DNA sequencing, and the correct overexpression strains finally stored at −80°C. The strains were incubated in LB medium at 37°C for 12 h with shaking at 220 rpm. The bacteria solution was then transferred to fresh medium. Bacteria were grown at 37°C to an OD_600_ value of between 0.6 and 0.8, and then isopropyl β-D-thiogalactoside (IPTG) was added at 28°C to induce protein expression. In the process of incubation and induction, 0.1 mmol/L CoCl_2_ was added to the LB medium, unless cobalt-free conditions were necessary (Pei et al., 2013). Protein expression was analyzed by sodium dodecyl sulfate–polyacrylamide gel electrophoresis (SDS-PAGE). Protein purification was performed using His-tag affinity chromatography (Rzeznicka et al., 2010).

### Activation of Proteins *in vitro*

The cobalt-free protein activation buffer consisted of 50 mmol/L phosphate-buffered saline (PBS) at pH 7.0, 10 μmol/L CoCl_2_, and 2 mmol/L DTT, unless otherwise noted. A CoCl_2_ solution was used as the cobalt donor. The final contents of Apo-AnhDEA and Holo-/Apo-AnhDA or Holo-/Apo-AnhEA in the buffer, and the concentrations of CoCl_2_ and DTT, were the same as those described by Zhou et al. (2009). These protein mixtures were incubated at 30°C for 12 h.

### Cobalt Content Determination

Proteins were dialyzed against 50 mmol/L PBS (pH 7.0) for 72 h at 4°C. After dialysis, the proteins were filtered through a 0.22-μm aperture filter membrane in preparation for subsequent analysis. The cobalt content in the proteins was detected using a Shimadzu atomic absorption spectrophotometer (AA-6300C) at a wavelength of 240.73 nm.

### Activity Assay and Enzyme Assay

The strains *E. coli*-Q4, Q5, and Q6 were overexpressed and grown to an OD_600_ value of 3.0. Bacteria solutions (5 mL) were then centrifuged at 8000 rpm for 20 min at 4°C. The sediments were washed with PBS three times, followed by the addition of 50 mmol/L PBS (pH 7.0) containing 0.1 mmol/L CoCl_2_ and 200 mg/L ACE. The activity of overexpressed strains was determined at 30°C for 10 min with shaking at 220 rpm.

The enzyme activity was determined at the optimal temperature and pH. The enzyme reaction system and one unit (U) of NHase activity were the same as previously described (Guo et al., 2019).

### UV-Vis Absorption Spectra

UV-Vis spectra of NHases were obtained with a BioTek SYNERGY H1 hybrid multi-mode microplate reader (BioTek Instruments, Inc, Vermont, United States) at spectrum scanning mode and room-temperature environment. NHases were dialyzed against 50 mmol/L PBS (pH 7.0) and then diluted to a concentration of 0.5 mg/ml.

### High-Performance Liquid Chromatography and Molecular-Exclusion Chromatography

ACE and its metabolite were analyzed using an Agilent 1200 HPLC system equipped with an Agilent G1314A UV detector (Agilent Technologies, Santa Clara, CA, United States). The HPLC mobile phase was a mixture of water (containing 0.01% acetic acid) and acetonitrile in a 70:30 ratio. The mobile phase flow rate was 1 mL/min.

Molecular-exclusion chromatography is usually used to estimate the molecular masses of purified proteins. In this study, a TSKgel G4000pwxl column (Tosoh Bioscience Ltd., Japan) equilibrated with 50 mmol/L PBS (pH 7.0) containing 0.2% NaN_3_ was connected to the Agilent 1200 HPLC system to isolate and purify proteins at a wavelength of 280 nm. The mobile phase flow rate was 0.5 mL/min. A Sigma-Aldrich gel filtration marker kit was used as the standard protein marker for column calibration. The eluate was collected using an automatic distribution collector.

### Molecular Docking

The structures of substrates ACE, THI, and IAN were obtained using Chem 3D software. The *Sc*NHase homology model was established in our previous study (Guo et al., 2019). The template (PDB code: 3qz5) was selected to establish the homology model for *Vb*NHase. The method for determining the homology model for *Vb*NHase was the same as described in our previous study (Guo et al., 2019). BSP-SLIM, a new method for ligand–protein blind docking using low-resolution protein structures, is useful for template-based coarse-grained algorithms in low-resolution ligand–protein docking and drug-screening (Lee and Zhang, 2012). The docking models were estimated by scores, with higher scores indicating better models. Protein–Ligand Interaction Profiler (PLIP) was used to identify interactions between proteins and their ligands. Model picture analysis was implemented using the Chimera program.

## Results

### ACE Degradation by Recombinant Strains

Activator proteins are necessary for NHase to promote NHase activity, and act as metal chaperones to aid cobalt ion insertion into α-subunits (Okamoto et al., 2010; Liu et al., 2012; Sun et al., 2017). According to bioinformatics analysis results, no activator protein was found to co-express with *Sc*NHase, and *Sc*NHase including only two β-subunit partial peptides (AnhD and AnhE) and an α-subunit (Guo et al., 2019). AnhD and AnhE were proved to be necessary to produce active *Sc*NHase (Guo et al., 2019). Therefore we next conducted an investigation to determine whether AnhD and AnhE in *Sc*NHase have activated protein functions.

**FIGURE 2:**
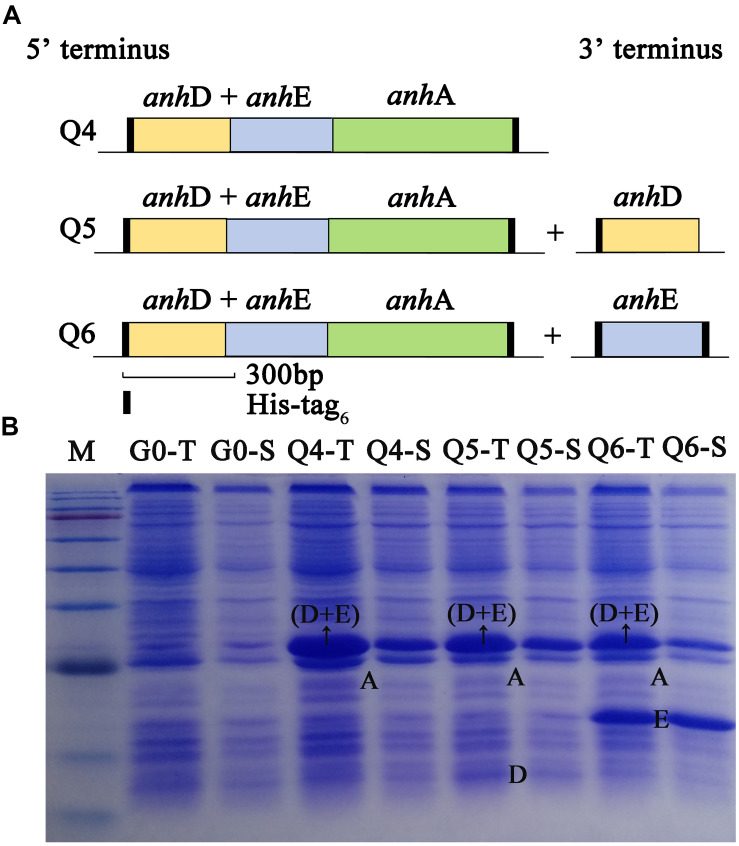
Construction and overexpression of recombinant strains of plasmid Q4 coexpressed with plasmid-AnhD and plasmid-AnhE. **(A)** Construction of recombinant strains of Q4, Q5, and Q6; **(B)** proteins of overexpressed recombinant strains detected by SDS-PAGE. G0-T and G0-S are the total protein and soluble protein for pET-28a(+) and pET-21a(+) without target gene overexpression in *E. coli* as a control. Lanes Q4-T, Q5-T, and Q6-T are total proteins for the corresponding strains. Lanes Q4-S, Q5-S, and Q6-S are soluble proteins for the corresponding strains. Lane M shows the standard protein markers (180.0, 140.0, 100.0, 80.0, 60.0, 45.0, 35.0, 25.0, 15.0, and 10.0 kDa).

**FIGURE 3:**
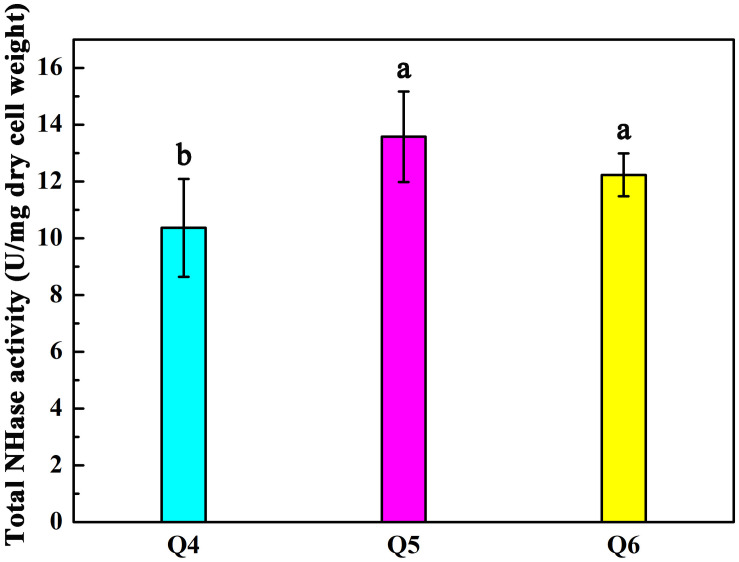
Activity of recombinant strains Q4, Q5, and Q6 transforming ACE. Triplicate independent experiments were conducted. Different superscripts (a and b) indicate significant differences at the *p* ≤ 0.05 level from one-way analysis of variance and Duncan’s test.

As shown in Figure 2A, the arrangement of *Sc*NHase subunits genes in plasmid Q4 was similar to the classical NHase gene structure (β-subunit and α-subunit genes). The activity of recombinant strain Q4 has been detected in our previous study (Guo et al., 2019). We assumed that AnhD and AnhE had functions as activator proteins, and attempted to co-express plasmid Q4 with plasmid AnhD and plasmid AnhE, namely plasmid Q5 and plasmid Q6, respectively (Figure 2A), which is similar to the classical NHase gene structure (β-subunit, α-subunit, and an activator protein). Figure 2B shows the protein expression of overexpressed plasmids Q4, Q5, and Q6. We used *E. coli* strains Q4, Q5, and Q6 to degrade ACE, with the activity of strains Q5 and Q6 found to be higher than that of strain Q4 (Figure 3), in agreement with our previous results. AnhD and AnhE subunits have a key amino acid site that plays a crucial role in *Sc*NHase activity (Guo et al., 2019). However, no significant difference was observed in the activity of strains Q5 and Q6 for the degradation of ACE. Therefore, the functions of AnhD and AnhE subunits in *Sc*NHase required further study.

### Activation of Apo-AnhDEA *in vitro* and Activity of ACE Transformation

In our previous study, we constructed recombinant *E. coli* strains Q0, Q1, Q2, and Q3 (Guo et al., 2019). Recombinant *E. coli* strains Q0, Q1, Q2, and Q3 were induced and overexpressed to produce the corresponding proteins AnhDEA, AnhA, AnhEA, and AnhDA. The proteins were purified using a nickel column and detected by SDS-PAGE (Supplementary Figure S1).

**FIGURE 4:**
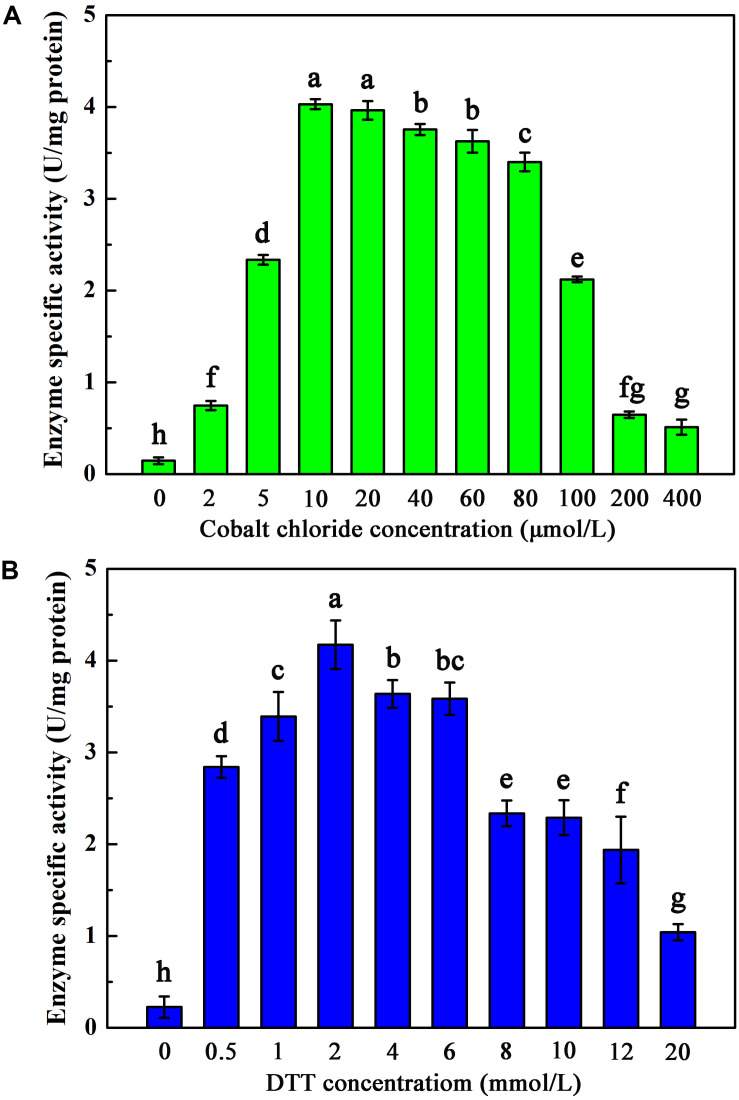
Activation of Apo-AnhDEA under various environment *in vitro*. **(A)** Effect of the cobalt chloride concentration on Apo-AnhDEA activation *in vitro*. 2 mmol/L DTT and 0, 2, 5, 10, 20, 40, 60, 80, 100, 200, or 400 μmol/L CoCl_2_ were added in activation buffer; **(B)** Effect of the DTT concentration on Apo-AnhDEA activation *in vitro*. 0, 0.5, 1, 2, 4, 6, 8, 10, 12, or 20 mmol/L DTT were added in activation buffer containing 10 μmol/L CoCl_2_. Triplicate independent experiments were conducted. Different superscripts (a, b, c, d, e, f, g, and h) represent significant differences at the *p* ≤ 0.05 level by one-way analysis of variance and Duncan’s test.

To investigate the maturation mechanism of AnhDEA *in vitro*, we detected the ACE transformation activity of AnhDEA induced and expressed under different conditions. AnhDEA induced under cobalt-added and cobalt-free condition were named holoprotein (Holo)-AnhDEA and apoprotein (Apo)-AnhDEA, respectively. We found the NHase activity of Apo-AnhDEA after incubated in activation buffer with CoCl_2_ and DTT added were significantly increased. Subsequently, the effect of different concentration cobalt on activation of Apo-AnhDEA *in vitro* was investigated and NHase activity of the resultant protein, R-apo-AnhDEA, was detected by HPLC method. The results showed that 10 μmol/L CoCl_2_ was the most appropriate concentration for Apo-AnhDEA activation *in vitro*, but no significant difference was observed in the NHase activity of R-apo-AnhDEA produced by activation buffer 10 and 20 μmol/L CoCl_2_ added (Figure 4A). When the final cobalt concentration in activation buffer was more than 20 μmol/L, the NHase activity of R-apo-AnhDEA had an apparent decrease (Figure 4A). Then the effect of different DTT concentration on vitro activation of Apo-AnhDEA in the presence of 10 μmol/L CoCl_2_ was conducted and the NHase activity of the corresponding R-apo-AnhDEA was measured. The results suggested 2 mmol/L DTT was the optimal concentration for Apo-AnhDEA activation *in vitro* (Figure 4B). The NHase activity of R-apo-AnhDEA increased by the DTT concentration increased in the range of 0 to 2 mmol/L, but a significant decrease was found when the DTT concentration was more than 4 mmol/L (Figure 4B). These findings indicated that activation buffer containing 10 μmol/L CoCl_2_ and 2 mmol/L DTT could provide the suitable condition for Apo-AnhDEA activation *in vitro*, and therefore it was used in the following experiments. And then more experiments about whether related proteins could be activated *in vitro* by activation buffer or not were conducted. Apo-AnhDEA incubated in activation buffer (50 mmol/L PBS containing 10 μmol/L CoCl_2_ and 2 mmol/L DTT) for 12 h produced the corresponding protein R-apo-AnhDEA. Apo-AnhDEA and Holo-AnhDEA incubated in 50 mmol/L PBS buffer containing 10 μmol/L CoCl_2_ for 12 h produced corresponding proteins Apo-AnhDEA+cobalt and Holo-AnhDEA+cobalt, respectively. The incubation treatment and analytical methods of AnhA, AnhDA, and AnhEA were the same as described above. As shown in Table 2, no significant difference was observed in the ACE transformation efficiency of Holo-AnhDEA and Holo-AnhDEA+cobalt, indicating that cobalt in Holo-AnhDEA was in a saturated state during cell induction, and that cobalt addition had no effect on its activity. Apo-AnhDEA showed almost no activity toward ACE, while R-apo-AnhDEA transformation activity for ACE was 35.98 ± 4.32% that of Holo-AnhDEA (Table 2). Furthermore, AnhA showed no activity for ACE conversion, while the activities of AnhDA and AnhEA for ACE conversion were very low. These results indicated that cobalt could insert into Apo-AnhDEA to form R-apo-AnhDEA bearing NHase function *in vitro* (added DTT). However, the activity of R-apo-AnhDEA activated *in vitro* was relatively low, indicating that other mature mechanisms of *Sc*NHase might be present.

**TABLE 2:** Activity of purified and treated AnhDEA, AnhDA, AnhEA, and AnhA for ACE transformation*^*b*^*.

**Protein**	**Relative activity (%)**
Holo-AnhDEA + Co	100.99 ± 5.96^a^
Holo-AnhDEA	100^a^
R-apo-AnhDEA	35.98 ± 4.32^b^
Apo-AnhDEA	4.63 ± 1.12^c^
Apo-AnhDEA + Co	3.30 ± 0.57^c^
Holo-AnhDA	0.21 ± 0.04^c^
R-apo-AnhDA	1.97 ± 0.27^c^
Apo-AnhDA	0.56 ± 0.13^c^
Holo-AnhEA	4.17 ± 0.29^c^
R-apo-AnhEA	0.71 ± 0.17^c^
Apo-AnhEA	0.14 ± 0.05^c^
Holo-AnhA	0
R-apo-AnhA	0
Apo-AnhA	0

*^*b*^Relative activity was calculated by dividing the specific enzyme activity by the largest specific enzyme activity. Activity of Holo-AnhDEA (11.82 U/mg) was defined as 100%. Values represent means ± S.D. for more than triplicate independent experiments. Different superscripts (a, b, and c) indicate significant differences at the p ≤ 0.05 level from one-way analysis of variance and Duncan’s test.*

### Cobalt Content in α-Subunits of Each Protein After Activation *in vitro*

To verify the consequence of cobalt insertion into *Sc*NHase, the cobalt contents of AnhDEA, AnhA, AnhDA, and AnhEA before and after different incubation treatments were detected by atomic absorption spectrometry. Cobalt was not detected in Apo-AnhDEA, Apo-AnhDA, Apo-AnhEA, and Apo-AnhA, but was detected in all proteins after incubating with activation buffer for 12 h (Table 3). Our results showed that one mole of R-apo-AnhDEA protein contained 0.66 ± 0.03 mol of cobalt, while one mole of Holo-AnhDEA protein contained 0.84 ± 0.10 mol of cobalt. No significant difference was observed in the cobalt contents per mole of R-apo-AnhDEA, R-apo-AnhDA, R-apo-AnhEA, and R-apo-AnhA (Table 3). These results further showed that cobalt could insert into the α-subunit of *Sc*NHase *in vitro*. However, significant differences in the cobalt content per mole were observed between Holo-AnhA and Holo-AnhDEA (Table 3). We speculated that another mechanism might exist to aid cobalt insertion into AnhA of *Sc*NHase *in vitro*. We assumed that AnhD and AnhE aided cobalt insertion into AnhA, and some related experiments were designed.

**TABLE 3:** Cobalt content of each purified protein before and after activation^*c*^.

**Protein**	**Cobalt content**
	***mol irons/mol protein***
Holo-AnhDEA	0.84 ± 0.10^a^
Apo-AnhDEA	0
R-apo-AnhDEA	0.66 ± 0.03^ab^
Holo-AnhDA	0.67 ± 0.11^ab^
Apo-AnhDA	0
R-apo-AnhDA	0.67 ± 0.15^ab^
Holo-AnhEA	0.58 ± 0.13^b^
Apo-AnhEA	0
R-apo-AnhEA	0.59 ± 0.10^b^
Holo-AnhA	0.57 ± 0.10^b^
Apo-AnhA	0
R-apo-AnhA	0.58 ± 0.11^b^

*^*c*^Values represent means ± S.D. for more than triplicate independent experiments. Different superscripts (a and b) indicate significant differences at the *p* ≤ 0.05 level from one-way analysis of variance and Duncan’s test.*

### Conversion of Apo-AnhDEA to Holo-AnhDEA in Holo-AnhDA and Holo-AnhEA

We assumed that either AnhD or AnhE acted as the activator protein. AnhDA and AnhEA were induced and expressed under cobalt-added and cobalt-free conditions. AnhDEA were induced and expressed under cobalt-free condition. Holo-AnhDA, Apo-AnhDA, Holo-AnhEA, Apo-AnhEA, and Apo-AnhDEA were then purified. To investigate Apo-AnhDEA conversion into Holo-AnhDEA in the presence of Holo-AnhDA or Holo-AnhEA, two experiments were conducted. The first combination was Apo-AnhDEA incubated with Holo-AnhDA or Holo-AnhEA for 12 h, and the second combination was Apo-AnhDEA incubated with Apo-AnhDA or Apo-AnhEA under activation buffer for 12 h. Subsequently we detected the efficiency of mixtures for ACE transformation. The ACE transformation efficiency of the Apo-AnhDEA and Apo-AnhDA mixture in activation buffer was 38.01 ± 1.33% of that of Holo-AnhDEA. The ACE transformation efficiency of the mixture of Apo-AnhDEA and Apo-AnhEA in activation buffer was 25.43 ± 1.58% of that of Holo-AnhDEA. These results indicated that cobalt can insert into Apo-AnhDEA in activation buffer, which was in agreement with the results for cobalt content above. The ACE conversion activity of Apo-AnhDEA and Holo-AnhDA after incubation for 12 h was 54.41 ± 1.35% of that of Holo-AnhDEA, while the ACE conversion activity of Apo-AnhDEA and Holo-AnhEA after incubation for 12 h was only 11.84 ± 1.66% of that of Holo-AnhDEA (see Table 4). According to the above results, Apo-AnhDEA was clearly activated by Holo-AnhDA and, therefore, showed the activity of NHase catalytic ACE. These findings showed that AnhDA has an important role for the conversion of Apo-AnhDEA and Holo-AnhDEA.

**TABLE 4:** Activity of protein mixtures of Apo-AnhDEA incubated with AnhDA and AnhEA^*d*^.

**Protein mixtures**	**Relative activity (%)**
Apo-AnhDEA + Apo-AnhDA +Cobalt + DTT	38.01 ± 1.33^b^
Apo-AnhDEA + Holo-AnhDA	54.41 ± 1.35^a^
Apo-AnhDEA + Apo-AnhEA +Cobalt + DTT	25.43 ± 1.58^c^
Apo-AnhDEA + Holo-AnhEA	11.84 ± 1.66^d^

*^*d*^Relative activity was calculated by dividing the specific enzyme activity by the largest specific enzyme activity. Activity of Holo-AnhDEA (11.82 U/mg) was defined as 100%. Values represent means ± S.D. for more than triplicate independent experiments. Different superscripts (a, b, c, and d) indicate significant differences at the *p* ≤ 0.05 level from one-way analysis of variance and Duncan’s test.*

### UV-Vis Absorption Spectra of NHases

**FIGURE 5:**
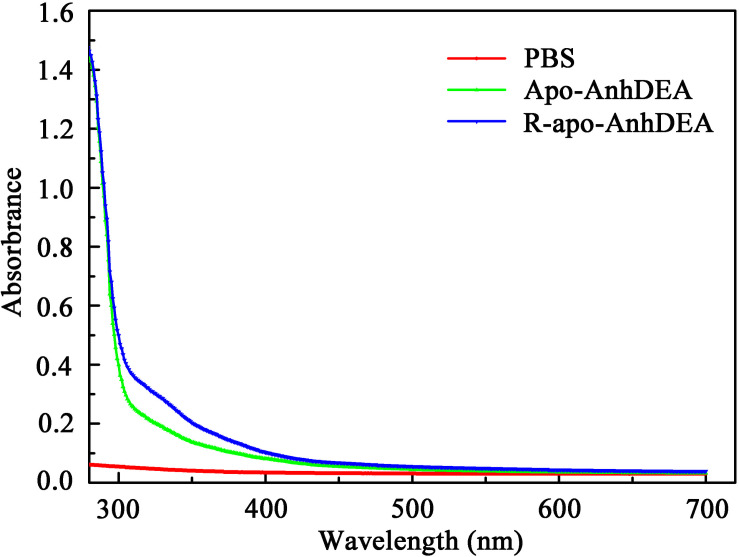
UV-vis absorption spectra of NHases.

To investigate the detailed character of cobalt ion insertion into Apo-AnhDEA and resultant R-apo-AnhDEA, we compared the property between Apo-AnhDEA and R-apo-AnhDEA by UV-Vis absorption spectra. The result of R-apo-AnhDEA showed an extra shoulder in the 300–350 nm region, which is distinct from Apo-AnhDEA (Figure 5).

### Protein Molecular Weight Determination

**FIGURE 6:**
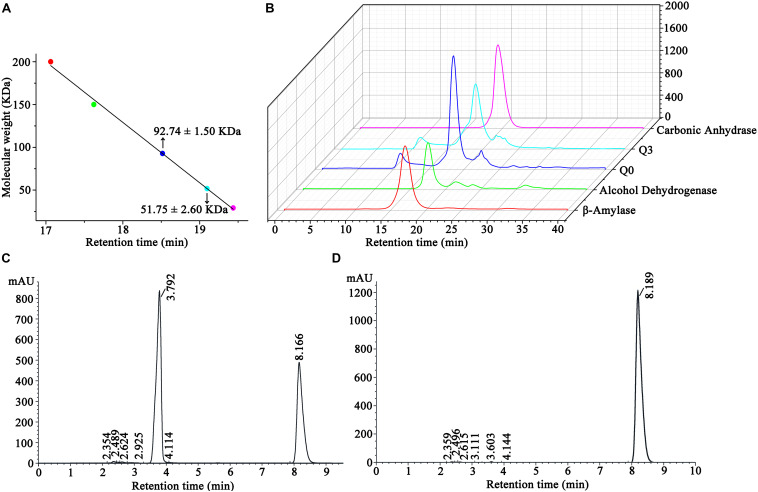
Molecular-exclusion chromatography analysis of molecular weight of *Sc*NHase and AnhDA in a nature state and HPLC analysis activity results of purified *Sc*NHase and AnhDA for ACE. **(A)** Calculation results of *Sc*NHase and AnhDA molecular weight; **(B)** molecular-exclusion chromatography of *Sc*NHase and AnhDA. Red, green, blue, cyan, and magenta represent β-amylase, alcohol dehydrogenase, *Sc*NHase, AnhDA, and carbonic anhydrase, respectively. HPLC results of ACE transformed by **(C)** *Sc*NHase and **(D)** AnhDA purified and collected by molecular-exclusion chromatography.

The molecular weights of *Sc*NHase and AnhDA in a natural state were also analyzed by molecular-exclusion chromatography. The results showed that the molecular weight of *Sc*NHase was 92.74 ± 1.5 kDa, while that of AnhDA was 51.75 ± 2.6 kDa (Figure 6A). The related spectrum is shown in Figure 6B, and the major peaks of proteins Q0 and Q3 were used to calculate the molecular weight. The protein filtered by molecular-exclusion chromatography was collected and used for ACE transformation. HPLC analysis showed that the collected *Sc*NHase can transform ACE (peak at a retention time of 8.166 min), producing a metabolite (IM-1-2) with a retention time of 3.792 min (Figure 6C), but ACE was not transformed by the collected AnhDA (Figure 6D). Our previous studies have verified three *Sc*NHase subunits corresponding to the SDS-PAGE band stained by Coomassie Brilliant Blue in MALDI-TOF MS and predicted that the molecular weight of the three subunits of AnhD, AnhE, and AnhA were 10.41, 11.35, and 21.33 kDa, respectively, while AnhDEA and AnhDA had a histidine tag of 3.3 kDa at the N-terminus (Guo et al., 2019). The α-subunit (AnhA) of AnhDA C-terminal carried a 1.06-kDa histidine tag. Therefore, we calculated and speculated that *Sc*NHase was a dimer (AnhD_2_E_2_A_2_), while AnhDA was in the form of AnhD_2_A in a natural state.

### Molecular Docking of *Sc*NHase and Substrate

**FIGURE 7:**
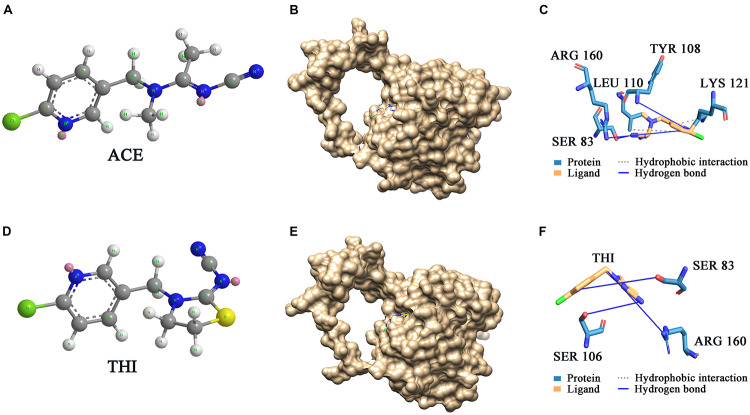
Docking results of *Sc*NHase with ACE and THI. **(A)** Structure of ACE; **(B)** surface of docking models between ACE and *Sc*NHase; **(C)** interaction between ACE and *Sc*NHase; **(D)** structure of THI; **(E)** surface of docking models between THI and *Sc*NHase; **(F)** interaction between THI and *Sc*NHase.

**FIGURE 8:**
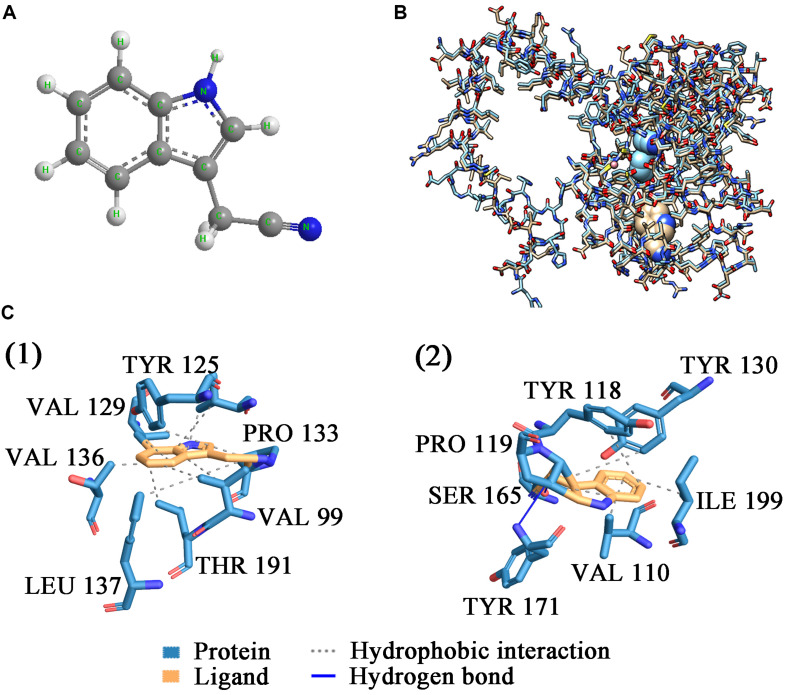
Docking results of *Sc*NHase from *S. canus* CGMCC 13662 and NHase from *V. boronicumulans* CGMCC 4969 with IAN. **(A)** Stick and ball figure of IAN; **(B)** comparison of docking results for *Sc*NHase from *S. canus* CGMCC 13662 (cyan) and NHase from *V. boronicumulans* CGMCC 4969 (steel gray) with IAN; **(C)** interaction between (1) *Sc*NHase and IAN, and (2) NHase from *V. boronicumulans* CGMCC 4969 and IAN.

*Sc*NHase hydrates ACE, THI, and BN, producing the corresponding amides, but does not hydrate IAN (Guo et al., 2019). However, NHase produced by *V. boronicumulans* CGMCC 4969 (*Vb*NHase) can hydrate IAN (Guo et al., 2019). The *Sc*NHase AnhA has a similarity of 45.07% with the *Vb*NHase α-subunit (Supplementary Figure S2). Therefore, we docked *Sc*NHase with ACE, THI, and IAN to investigate the interactions. A homology model for *Sc*NHase has been developed (Guo et al., 2019). We used BSP-SLIM to screen the binding sites of *Sc*NHase with the substrates. The docking scores of *Sc*NHase with ACE, THI, and BN were higher, indicating that the models were better. The optimal positions of ACE and THI docking with *Sc*NHase were exactly located at the *Sc*NHase active center close to the cobalt ion, and were in the same solvent-accessible surface pocket of *Sc*NHase (Figure 7). The interaction between protein (*Sc*NHase) and substrate ACE comprised a hydrogen bond and hydrophobic interaction, with SER83, TYR108, and ARG160 were involved in the hydrogen bond and LEU110 and LYS121 involved in the hydrophobic interaction. SER83, TYR106, and ARG160 in *Sc*NHase were involved in the hydrogen bond with THI. We also developed a homology model for NHase from *V. boronicumulans* CGMCC 4969 (Supplementary Figure S3). The molecular model alignment of substrate IAN (Figure 8A) docking with *Vb*NHase and *Sc*NHase is shown in Figure 8B. Interestingly, the optimal pose of ligand IAN was located close to the active center of *Vb*NHase. The optimal pose of ligand IAN was far from the active center of *Sc*NHase. According to PLIP analysis, the interaction between amino acids of *Sc*NHase and substrate IAN was mainly hydrophobic, but a hydrogen-bond interaction also existed between amino acids of *Vb*NHase and substrate IAN (Figure 8C).

## Discussion

Zhou et al. (2009) found that Apo-α_2_β_2_ had almost no NHase catalytic activity, while R-apo-α_2_β_2_ had only 5.97% of the activity of Holo-α_2_β_2_. In contrast, in the present study, the NHase catalytic activity of R-apo-AnhDEA was 35.98 ± 4.32% of that of Holo-AnhDEA. Zhou et al. (2009) showed that the cobalt content per mole of Holo-α_2_β_2_ was 0.88 ± 0.03 mol, while that of R-apo-α_2_β_2_ was only 0.16 ± 0.03 mol. In our study, the cobalt content per mole of Holo-AnhDEA was 0.84 ± 0.10 mol, while that of R-apo-AnhDEA protein was 0.66 ± 0.03 mol. And there was an extra shoulder in the 300–350 nm region in UV-Vis spectra of R-apo-AnhDEA comparing to Apo-AnhDEA, which is similar to the results of Zhou et al. (2008) and Pei et al. (2013). This extra shoulder region reflecting the S→Co^3+^ charge transfer was recognized as a characteristic of active Co-type NHase (Zhou et al., 2008; Pei et al., 2013). These results strongly suggested that cobalt can insert into AnhA of Apo-AnhDEA, with the resulting R-apo-AnhDEA showing NHase activity.

Studies have shown a unique ligation mode between cobalt and peptides in Co-type NHase active centers (Miyanaga et al., 2001; Zhou et al., 2009; Nelp et al., 2014). Metal ion insertion into the NHase active center causes the post-translational modification of cysteine residues, which cannot be observed in apoenzyme NHase (Kuchar and Hausinger, 2004; Miyanaga et al., 2004; Zhou et al., 2008). Post-translational oxidative modification of cysteine residues in the active center [CXLC(SO_2_H)SC(SOH)] is crucial for NHase catalytic activity (Murakami et al., 2000; Hashimoto et al., 2008; Martinez et al., 2014). As an active site, the cysteine-sulfinate oxygen in the amino acid residues of nitrile hydratase α-subunits can provide a scaffold site to activate water molecules to attack metal-binding nitriles or as nucleophiles (Hashimoto et al., 2008; Martinez et al., 2014; Nelp et al., 2014). It was reported that activator protein is essential for both post-translational cysteine oxidation and cobalt insertion into the Co-type NHase (Zhou et al., 2009; Liu et al., 2012). However, there are also some different results. Pei et al. (2013) found that the recombinant Co-type NHase from *Aurantimonas manganoxydans* (*Am*NHase) could express successfully in *E. coli* and cobalt could incorporate into cobalt-defective apoenzyme (Apo-) *Am*NHase and regenerate to active *Am*NHase without an activator accessory protein complex *in vitro*. A reduction environment (β-merceptoethanol) can promote the activity regeneration of Apo-*Am*NHase *in vitro* by breaking a key disulfide bond thereby promoting insertion of cobalt into the apoenzyme (Pei et al., 2013). Similarly, this phenomenon also occurred in Fe-type NHase. A functional Fe-type NHase from *Comamonas testosteroni* Ni1 (*Ct*NHase) were successfully co-expressed in *Escherichia coli* (*E. coli*) without its activator protein by Kuhn et al. (2012). Researchers investigated the structure of Fe-type NHase, *Ct*NHase and found that Fe (III) ion in the active center is solvent exposed, which may provide a much more direct route for metal ion insertion (Kuhn et al., 2012). Interestingly, there are specific activator protein genes corresponding to *Am*NHase and *Ct*NHase in their respective genomes, but no activator protein genes were found in *S. canus* CGMCC 13662 genome (Guo et al., 2019). *Sc*NHase including three subunits without activator protein is absolutely different from all NHase mentioned above in structurally, which suggested that the maturation mechanism of *Sc*NHase is different from that of them. Nelp et al. (2014) studied a three-subunit nitrile hydratase (TNHase) derived from *Streptomyces rimosus*, with the results showing that the α-subunit alone of TNHase (ToyJ) was sufficient for NHase catalytic activity and to synthesize the active site complex with full post-translational modifications. The results in our previous study suggested that *Sc*NHase maturation mechanism is different from TNHase (Guo et al., 2019). Therefore the maturation mechanism of *Sc*NHase without activator protein is worthy of further study. In this study, we found that cobalt can insert into AnhA of *Sc*NHase in the absence of activator protein under DTT added vitro environment, which is similar to the results of Pei et al. (2013). At the same time, we investigated the function of subunits in *Sc*NHase.

Previous studies have reported that cobalt can be inserted into apo-NhlAE, apo-NhhAG, and apo-α(P14K)_2_ instead of the α-subunit in apoenzyme NHase under an *in vitro* reduction environment (dithiothreitol, 2-mercaptoethanol, or glutathione). Furthermore, NhlE, NhhG, and P14K assist with cobalt insertion into α-subunits, which are essential for the conversion of apo-α to holo-α *in vitro* (Zhou et al., 2009; Zhou et al., 2010; Liu et al., 2012). Zhou et al. (2009) found that the NHase activity was activated when apo-α_2_β_2_ was incubated with holo-αe_2_, and that NhlE, as the self-subunit swapping chaperone and metal chaperone, was necessary for L-NHase maturation. However, our results indicated that the NHase activity increased to 54.41 ± 1.35 of that of Holo-AnhDEA when Apo-AnhDEA was mixed with Holo-AnhDA for 12 h of incubation. The NHase activity increased to 38.01 ± 1.33 of that of Holo-AnhDEA when Apo-AnhDEA was mixed with Apo-AnhDA for 12 h of incubation in a reduction environment of cobalt and DTT. These findings showed that Holo-AnhDA can activate Apo-AnhDEA, with resultant R-apo-AnhDEA showing NHase activity. AnhDA was verified to be in the form of AnhD_2_A, a heterotrimer complex, that is similar to NhlAE, NhhAG, and α(P14K)_2_ (Zhou et al., 2009, 2010; Liu et al., 2012). These results conformed to self-subunit swapping, with cobalt ions tightly binding to AnhA, cobalt-containing AnhA in Holo-AnhD_2_A exchanging with cobalt-free AnhA in Apo-AnhDEA, and the resultant AnhDEA showing NHase activity. AnhD_2_A showed functions as a swapping chaperone and metal chaperone, similar to NhlAE (αe_2_) (Zhou et al., 2009).

The *anhD* gene is located upstream of *anhE* and *anhA* genes in *Sc*NHase, and AnhD and AnhE are homologous with the N-terminus and C-terminus of the NHase β-subunit, respectively (Guo et al., 2019). Many studies have reported that self-subunit swapping chaperone sequences have a weak similarity with NHase β-subunits (Wu et al., 1997; Zhou et al., 2009, 2010; Okamoto et al., 2010; Liu et al., 2012). Liu et al. (2012) showed that self-subunit swapping does not rely on the gene order of the α-subunit and β-subunit. This evidence further supported that the AnhD subunit was not only a part of the β-subunit, but also acted as a self-subunit swapping chaperone and metal chaperone to aid cobalt insertion into the *Sc*NHase α-subunit and *Sc*NHase maturation. Interestingly, AnhD was identified as the N-terminus of the β-subunit (Guo et al., 2019), which was speculated to be associated with the evolution strategy of NHase. Further evidence is needed to support this hypothesis.

**FIGURE 9:**
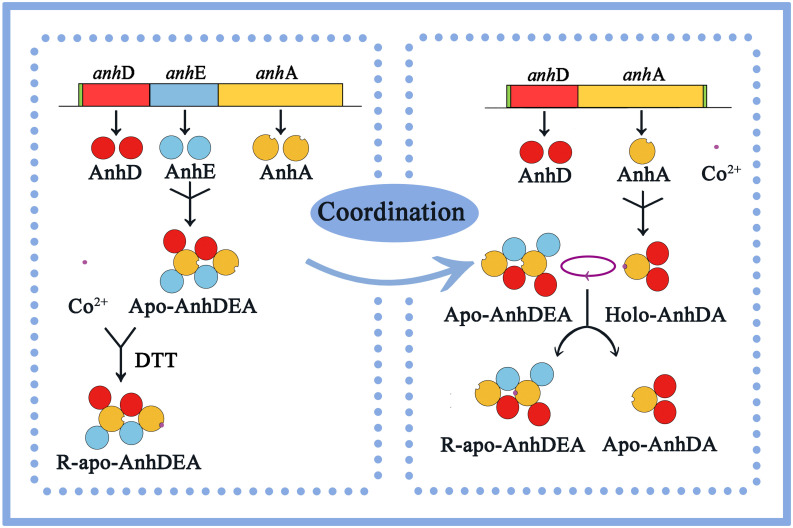
Proposed plausible model for cobalt incorporation into *Sc*NHase.

In this study, we concluded that cobalt was inserted into the α-subunit of Apo-AnhDEA *in vitro* by direct insertion in the absence of activator protein and coordinated self-subunit swapping assisted by AnhD as a metal ion chaperone and self-subunit swapping chaperone. Considering all the above results and speculation, we have proposed a plausible model for the process of cobalt incorporation into *Sc*NHase *in vitro*. As shown in Figure 9, as *Sc*NHase is a dimer (AnhD_2_E_2_A_2_) in the natural state and folds into a specific structure, allowing cobalt to insert into one AnhA of Apo-AnhD_2_E_2_A_2_ in reductive (DTT added) environment, resulting in R-apo-AnhD_2_E_2_A_2_. Cobalt-containing AnhA in Holo-AnhD_2_A can replace another cobalt-free AnhA in Apo-AnhD_2_E_2_A_2_. Subunit AnhD is necessary for *Sc*NHase post-translational maturation *in vitro*. To our knowledge, this is the first report of the post-translational maturation mechanism of a NHase with three subunits in the absence of activator protein.

Several laboratories have verified that NHase has a good structural correlation with function (Mascharak, 2014). Active site activation is the first step of NHase catalysis of the substrate, and the optimal position of substrate acrylonitrile docking with NHase from *Pseudonocardia thermophila* JCM 3095 (*Pt*NHase) is exactly located at the active center of *Pt*NHase, close to the cobalt ion center (Yu et al., 2008). A hydrogen-bonding network associated with water had a stable role in the active site structure, narrowing it from the entrance channel to the metal site, which might be related to the substrate specificity of NHase (Nakasako et al., 1999; Mascharak, 2014). In the model of ligand IAN docked with *Sc*NHase, the optimal docking pose was far from the active center of *Sc*NHase, and only weak hydrophobic interactions existed between *Sc*NHase and IAN. In contrast, strong hydrogen bonds were present between ligand and protein from the results of ACE and THI docked with *Sc*NHase, which were beneficial for protein–ligand complex stability, resulting in a stronger affinity. Therefore, this represents another example of NHase function correlating to structure, with the affinity between *Sc*NHase and IAN determined by the structure of *Sc*NHase. Further research on the *Sc*NHase structure is still needed.

In our previous study, we found the *Sc*NHase was similar to eukaryotic NHase according to bioinformatics analysis, with no activator protein genes recognized in their genome (Guo et al., 2019). The mechanism of cobalt insertion into *Sc*NHase *in vitro* and substrate specificity could be instructive for research into eukaryotes NHase. Furthermore, the structural character and substrate specificity of *Sc*NHase was drastically different to that of NHase applied for biodegradation in previous research reports. Therefore, *Sc*NHase could be further developed to transform other nitrile-containing contaminants and has good prospects for environmental remediation.

## Conclusion

We have detected the activity of R-apo-AnhDEA for ACE transformation, indicating that Apo-AnhDEA was activated *in vitro*. The cobalt contents in the related proteins verified that cobalt was able to insert into AnhA. *Sc*NHase and AnhDA in the natural state were in the form of AnhD_2_E_2_A_2_ and AnhD_2_A, respectively. Holo-AnhD_2_A activated the NHase catalytic activity of Apo-AnhD_2_E_2_A_2_, indicating that self-subunit swapping occurred between cobalt-containing AnhA in Holo-AnhD_2_A and cobalt-free AnhA in Apo-AnhD_2_E_2_A_2_. AnhD has functions of *Sc*NHase β-subunit, and also acts as a metal ion chaperone and self-subunit swapping chaperone for *Sc*NHase post-translational maturation. However, we found no similar function in AnhE. We have proposed for the first time that *Sc*NHase maturation *in vitro* relies on direct cobalt insertion without activator protein and coordinated self-subunit swapping mechanism. Based on the results of substrate docking with *Sc*NHase, only a weak hydrophobic interaction between protein and IAN was identified. *Sc*NHase showed almost no affinity with IAN, unlike ACE and THI, which was determined by the *Sc*NHase structure. This study provides a theoretical basis for future research on the maturation mechanism of NHase with three subunits.

## Data Availability Statement

All datasets generated for this study are included in the article/Supplementary Material.

## Author Contributions

LG designed and conducted the experiments, prepared most figures, and wrote the manuscript. Y-JD supervised the research. XC and H-YJ assisted LG to revise the manuscript. All authors contributed to the article and approved the submitted version.

## Conflict of Interest

The authors declare that the research was conducted in the absence of any commercial or financial relationships that could be construed as a potential conflict of interest.
