# Supplementary material for: Maturation Mechanism of Nitrile Hydratase From Streptomyces canus CGMCC 13662 and Its Structural Character
Source: Front Microbiol. 2020 Jun 25;11:1419. doi: 10.3389/fmicb.2020.01419 (PMC7329996; doi:10.3389/fmicb.2020.01419)
Supplement: Supplementary file 1 [file Data_Sheet_1.docx]

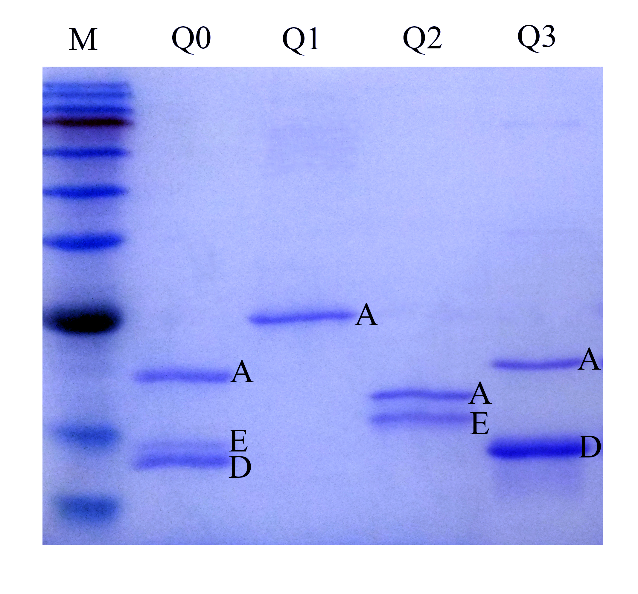


**FIGURE S1 | Sodium dodecyl sulfate–polyacrylamide gel electrophoresis (SDS-PAGE) analysis of purified proteins from recombinant strains.** Lane M shows the standard protein markers (180.0, 140.0, 100.0, 80.0, 60.0, 45.0, 35.0, 25.0, 15.0, and 10.0 kDa). Labels A, D, and E indicate expressed AnhA, AnhD, and AnhE, respectively.


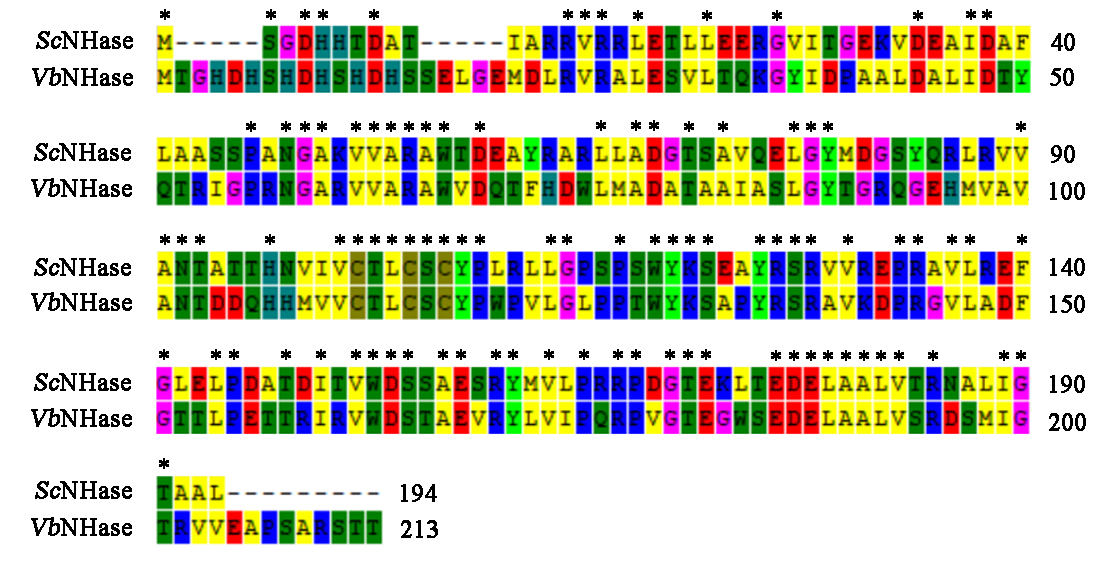


**FIGURE S2 | Alignment result of *Sc*NHase and *Vb*NHase.** The mark “*” represents conserved amino residues. Different colors were based on biochemical properties of residues, which was conducted by software Mega 6.06.


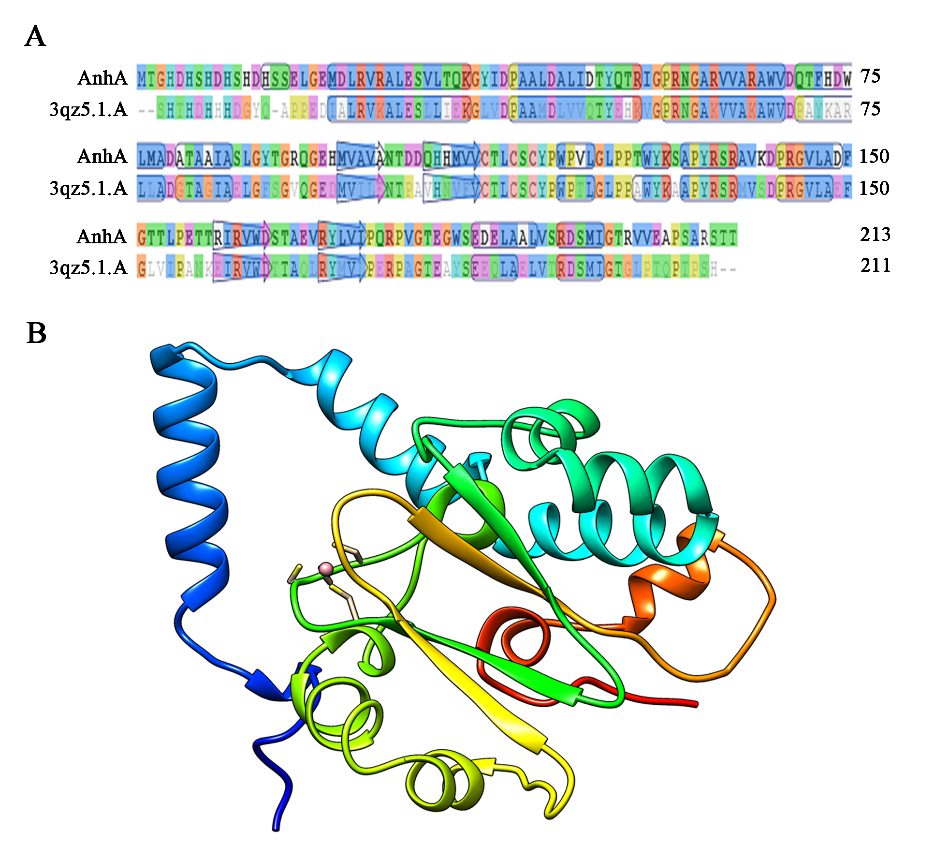


**FIGURE S3 |** **Homology model for** ***Variovorax boronicumulans* CGMCC 4969 (*Vb*) NHase and related analyses.** (A) Alignment of *Vb*NHase and model template NHase; (B) homology model of *Vb*NHase.
